# A large outbreak of COVID-19 linked to an end of term trip to Menorca (Spain) by secondary school students in summer 2021

**DOI:** 10.1371/journal.pone.0280614

**Published:** 2023-02-03

**Authors:** Lluís Forcadell-Díez, Cristina Rius, Raúl Salobral, Nacho Sánchez-Valdivia, Jacobo Mendioroz, Pere Godoy, Elisabet Badiella Jarque, David Ferrandiz-Mont, Daniel Moreno Cervera, Berta Jordan Suriñach, Alba Vilalta-Carrera, Víctor Guadalupe-Fernández, Julia Mateo Basilio, Sergi Farràs Tapiol, Gloria Pérez

**Affiliations:** 1 Agència de Salut Pública de Barcelona, Barcelona, Spain; 2 Universitat Pompeu Fabra, Barcelona, Spain; 3 CIBER de Epidemiología y Salud Pública, Madrid, Spain; 4 Institut d’Investigació Biomèdica Sant Pau (IIB Sant Pau), Barcelona, Spain; 5 Subdirecció General de Vigilància i Resposta a Emergències de Salut Pública, Agència de Salut Pública de Catalunya, Generalitat de Catalunya, Barcelona, Spain; Hospital Universitat de Sant Joan, Universitat Rovira I Virgili, SPAIN

## Abstract

**Background:**

An outbreak of severe acute respiratory syndrome coronavirus 2 (SARS-CoV-2) occurred in young people from Catalonia (Spain) who travelled to Menorca (Spain) in summer 2021. This outbreak appeared when governments relaxed Covid-19 preventive measures: the mask usage and the opening of nightlife. It was related to a super-disseminating mass event: Sant Joan festivities in Ciutadella. The aim of this article is to describe an outbreak of COVID-19 in young people aged 17–19 years from Catalonia travelling to Menorca.

**Methods:**

This is an observational study of a COVID-19 outbreak. The study population comprised Catalonian youth aged 17–19 years who travelled to Menorca from 15 June to 10 July. Epidemiological descriptive indicators were obtained. Descriptive and geographical statistics were carried out. Bivariate Moran’s I test was used to identify spatial autocorrelation between the place of residence and deprivation. The outbreak control method was based on identifying and stopping chains of transmission by implementing the test-trace-isolate-quarantine (TTIQ) strategy.

**Results:**

We identified 515 confirmed cases infected in Menorca, 296 (57.5%) in girls and 219 (42.5%) in boys, with a total of 2,280 close contacts. Of them, 245 (10.7%) were confirmed as cases. The cases were diagnosed between 15 June and 10 July. None of the persons with confirmed infection died or required hospitalisation. The attack rate was 27.2%. There was an inverse relationship between deprivation and number of confirmed cases (p<0.005), there were clusters of confirmed cases in the most socioeconomic favoured areas.

**Discussion:**

The outbreak is related with young people from socioeconomic favoured areas who travelled to Menorca in summer 2021. Failure to comply with preventive measures in binge-drinking events and during holidays may have favoured SARS-CoV-2 transmission. The interauthority coordination and establishment of a clear line of leadership allowed continuous communication between institutions, which were key to managing this complex COVID-19 outbreak.

## Introduction

**Fig 1:**
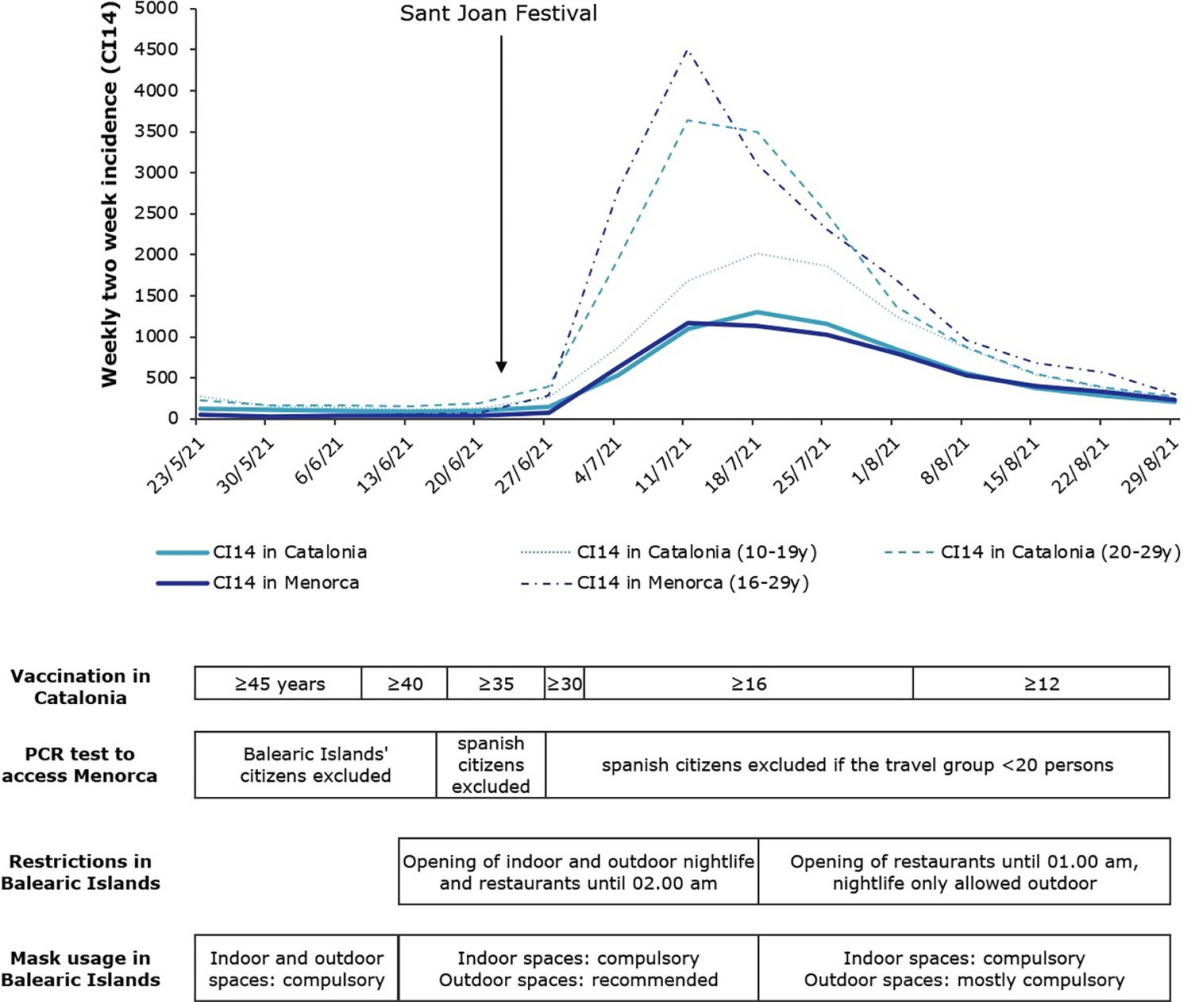
Evolution of weekly two-week incidence (CI14) in Catalonia and Menorca per 100 000 in summer 2021 and evolution of selected policies to control Covid-19 pandemics. Data for Catalonia was obtained from public repository https://dadescovid.cat; Data for Menorca was obtained from public repository https://www.ibsalut.es/coronavirus-covid-19/situacio-actual-de-la-covid-19-a-les-illes-balears. Covid-19 preventive measures were obtained from the Butlletí Oficial de les Illes Balears http://www.caib.es/boib/.

At the end of epidemiological week 23, which ended on June 13, 2021, transmission of severe acute respiratory syndrome coronavirus 2 (SARS-CoV-2), causing coronavirus disease 19 (COVID-19), had been decreasing both in Catalonia and in Europe from April. In Catalonia, the two week incidence (CI14) was 93 new cases per 100,000 inhabitants [1], while in Europe it was 72 per 100,000, national range 9–154 [2]. By age group, the CI14 was 118 per 100,000 inhabitants among 10–19 year-olds and 157 per 100,000 inhabitants among 20–29 year-olds (Fig 1). On 13 June, 43.3% of the population in Catalonia had received at least one COVID-19 vaccine dose, and 26.6% were fully vaccinated, with 2 doses [3]. The percentage of positive reverse transcriptase-polymerase chain reaction (RT-PCR)/rapid antigen tests (RAT) was 3.8%. Only 4 weeks later, the CI14 in Catalonia was among the highest in Europe at 1,092 per 100,000. The CI14 was 1,684 per 100,000 inhabitants in the group aged 10–19 years and was 3,640 per 100,000 in people aged 20–29 years (Fig 1).

The end-of-year trip is a long-awaited moment for all secondary school students. In Catalonia, the trips take place at the end of the school year in the last years of secondary school, ISCED-4, in the International Standard Classification of Education [4]. Trips are usually organized after the university entrance exams at the beginning of June. A typical destination is Menorca (Balearic Islands, Spain), a medium-sized island with 95,641 inhabitants [5]. The most important festivities in Menorca (the night of Sant Joan) take place on 23 June, in Ciutadella, when tens of thousands of people gather at beaches and clubs. These festivities are usually attended by young people from Catalonia. Many of them usually arrive a few days previously. In the context of the COVID-19 pandemic, the Sant Joan festivities were cancelled on April 1 [6]. In June, the epidemiological situation in Menorca was favourable, with a CI14 of 36. On 15 June, the Government of the Balearic Islands authorized the opening of nightlife and restaurants until 02.00 am (Fig 1). It also extended capacity to 75% of full capacity if masks were worn. There was no limit on social and family gatherings. Restrictions at ports and airports included passport control for national and international travellers [7].

Epidemiological surveillance in Catalonia is organised through the Subdirectorate General for Public Health Emergency Surveillance and Response of the Public Health Agency of Catalonia (ASPCAT). This institution has central services, in contact with their stakeholders at the national level, and different local services, which carry out field epidemiology. In the city of Barcelona, this role is carried out by the Barcelona Public Health Agency (ASPB), which acts as the health authority. In outbreaks that territorially exceed a local service, the epidemiological investigation is conducted from the place where the transmission occurred, or from the place with the highest number of cases. The usual coordination mechanisms between local and central services include a weekly inter-territorial meeting and additional technical coordination if necessary.

On Wednesday, 23 June, 2021 several primary healthcare centres in the city of Barcelona identified a total of 21 cases of COVID-19 in young people. The centres quickly reported the aggregation of cases to the ASPB, in the city, and stated that transmission may have been encouraged by a trip. In one of the centres, 3 cases with epidemiological links were confirmed and the affected individuals reported they had been infected during an end-of-year trip to Menorca [8]. They were the first young people returning from Menorca. At the same time, other local services, such as Tarragona, Vallès and Girona, had already detected cases of COVID-19 in young people from Menorca. When the ASPB reported the outbreak, the epidemiological link was quickly found with cases from other local services.

The aim of this article is to describe an outbreak of COVID-19 in young people aged 17–19 years from Catalonia travelling to Menorca. Study of this outbreak allows us to broaden our knowledge of the social factors favouring transmission of COVID-19, particularly in young people.

## Methods

### Study design and definitions of cases and close contacts

This is an observational study of a COVID-19 outbreak [9].

We defined a suspected case as any person with a clinical manifestation of an acute respiratory infection of sudden onset of any severity that presents, among others, fever, cough, or shortness of breath. Other symptoms such as rhinorrhoea, nasal congestion, sternness, headaches, odynophagia, anosmia, agenesis, muscular pain, diarrhoea, chest pain or headaches, among others, may also be considered symptoms of suspected SARSCoV-2 infection according to clinical criteria. SARS-CoV-2 testing was performed on oropharyngeal swabs taken by healthcare workers. Both the RAT and RT-PCR were considered valid diagnostic tests. However, in one suspected case with a negative RAT result, RT-PCR was performed [8]. The swabs were analysed by authorised laboratories. These authorised laboratories recorded the results of these tests and reported either confirmed cases or genome sequence results to the public health authorities.

We defined a confirmed case as a person with a positive RT-PCR or RAT test result for SARS-CoV-2 [8]. Thus, only those persons with positive diagnostic tests were considered confirmed cases. Confirmed cases were defined as symptomatic if they had fever, cough, or shortness of breath and as asymptomatic if they had no symptoms. The inclusion criteria for the consideration of primary confirmed case in this outbreak were diagnosis between 15 June and 10 July, age between 17 and 19 years, and a history of travel to Menorca. A wide time interval was established to avoid loss of cases.

We defined a close contact as any person who had been in the same location as a primary case, within 2 metres´ distance, and for a total cumulative time of 15 minutes or more, within 24 hours of the 48 hours before symptom onset in symptomatic individuals or before a positive test result in asymptomatic persons [8]. Mask use was not considered in the determination of close contacts due to young people´s poor adherence to this preventive measure in leisure and festive settings. We defined a secondary confirmed case as any person who was in close contact with a confirmed primary case with a positive RT-PCR or RAT test result and who did not travel to Menorca.

### Study population and management of cases and close contacts

**Fig 2:**
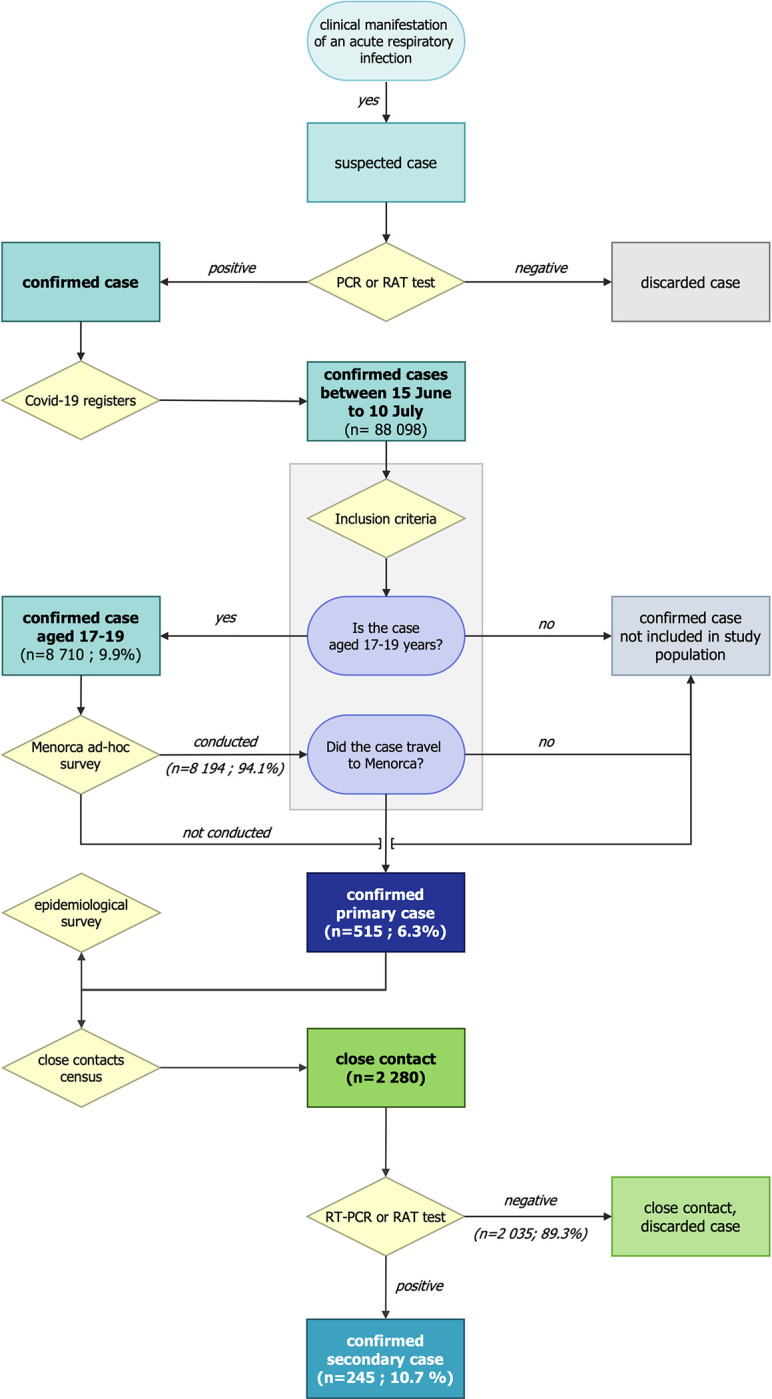
Classification and management of cases and its close contacts.

The study population comprised Catalonian youth aged 17–19 years who travelled to Menorca from 15 June to 10 July. To obtain the study population, we carried out an extraction of COVID-19 registers of the Catalonian Department of Health. During the study period, confirmed cases occurred in 8,710 youth aged 17–19 years (Fig 2). These youth were contacted by telephone to identify those who were in Menorca. The telephone calls were carried out by Public Health Case Agents, people trained to conduct both epidemiological surveys (S1 and S2 Appendices) and contact tracing [10]. The agents also explained how to reduce the risk of transmission to cohabitants, including cleaning, ventilation, and room segregation measures. Of the total number of young people, 8,378 (94.1%) could be contacted. In 332 cases (3.8%), no telephone contact was made, as the number was not available or was not taken; thus, they were excluded from the study. Among those contacted, 515 (6.3%) had travelled to Menorca. They were the primary cases in our study. A total of 2,280 close contacts were identified. They were telephoned by Public Health Close Contacts Agents to schedule RT-PCR tests at their primary healthcare centres and to recommend quarantine measures. Among the close contacts, 245 (10.7%) were confirmed as secondary cases. The same agents followed up close contacts and control measures on days 7 and 10 from the last date of contact with the confirmed case [10].

### Outbreak analysis and information sources

Descriptive statistics were carried out. Absolute and relative frequencies were calculated to describe the sociodemographic characteristics of confirmed cases. The main variable was being a confirmed case. Explanatory variables consisted of sociodemographic variables (age, sex, and place of residence), case criteria (primary or secondary), the test performed (RT-PCR or RAT), symptoms (symptomatic, asymptomatic), episode (first COVID-19 infection or reinfection), trip planning (self-organised or travel agency), accommodation (tourist apartment rental, private house, hotel, tourist complex or rural house), and attendance at parties (yes or no and if yes what kind of parties).

The attack rate was calculated by dividing the number of cases by the sum of cases and its close contacts. People who were close contacts of 2 or more cases were considered as one. Epidemiological curves were traced. The effect of the outbreak on the evolution of Covid-19 in Catalonia was approximated by calculating the percentage of cases of this outbreak with respect to the total number of young people in the affected age group. All analyses were stratified by sex.

Confirmed cases were georeferenced and assigned to their primary healthcare areas for geographical analysis. We use the incidence as dependent variable and the Composite socioeconomic index (CSI). The CSI is an index of deprivation used to assign the budgets of the primary healthcare areas in Catalonia (Spain) and is valid both in urban and rural areas [11]. Descriptive geographical analysis was carried out using Choropleth maps. We carried out a cluster analysis and we tested the spatial autocorrelation using univariate and bivariate Moran’s I test with 199 permutations.

Individuals’ sociodemographic variables and vaccination status were obtained from Central Health Registry of the Catalonian Health Service (Table 1). Clinical variables, risk factors and social needs were obtained from an epidemiological survey [10] (S1 Appendix). Transport to the island, accommodation, travel arrangements, attendance at parties and follow-up of preventive measures during the trip were obtained from an ad-hoc epidemiological survey of Menorca (S2 Appendix), conducted in response to this outbreak.

**Table 1:** Data sources during investigation of the Menorca trip outbreak of SARS-CoV-2. Catalonia, June, and July of 2021.

Source	Information available
Central Health Registry of the Catalan Health Service	• Personal information (age, sex, nationality, place of residence)
	• Ecological information of the area of residence (composite socioeconomic index)
	• Information on vaccination status
COVID-19 registers of the Catalonian Department of Health	• Day of consultation
	• Presence of symptoms
	• Day of symptom onset
	• RAT test results and notification of confirmed cases
Authorised laboratories	• RT-PCR results and notification of confirmed cases
	• Genome sequencing
Epidemiological survey	• Clinical course and symptoms
	• Risk factors
	• Social needs and need for proper isolation
Ad-hoc epidemiological survey of Menorca	• Transport to the island
	• Accommodation on the island
	• Travel arrangements
	• Activities during the trip
	• Follow-up of preventive measures
	• Name of travel agency

Epidemiological survey is included in S1 Appendix. Ad-hoc epidemiological survey of Menorca is included in S2 Appendix.

Statistical analyses were carried out using the statistical software STATA v15. Geographical analyses were carried out using the geographical software GeoDa. Choropleth maps were obtained using Datawrapper tool. Figures were created using Excel and PowerPoint software.

### Qualitative analysis

A qualitative phenomenological study [12] was conducted to understand the young people’s perceptions of the risk and transmission of COVID-19 in the context of the outbreak, the activities that may have encouraged transmission, and the organisation of the trip. The sample was obtained through convenience sampling, augmented by the snowball strategy. In addition to the epidemiological surveys, 13 interviews were conducted with confirmed cases aged 17–18 years who had a history of travel to Menorca. Interviews were conducted by telephone by researchers used to dealing with young people [13]. These interviews were not recorded; the most relevant aspects of each were summarised in a research notebook. The researchers conducted an interpretative thematic content analysis. The young people were guaranteed the confidentiality of their contributions and oral consent was requested to proceed with the interviews.

### Outbreak control measures

Due to the size of the outbreak, the setting, and the epidemiological situation in Catalonia, the outbreak investigation was conducted under time and resource constraints. We followed the testy-trace-isolate-quarantine (TTIQ) intervention strategy [14], giving priority to screening and isolation of suspected cases of COVID-19, as well as tracing, quarantine and testing of close contacts (Table 2). This strategy was complemented by enhanced surveillance, an active search for cases possibly involved in the outbreak, and the identification of exposures and activities conducive to transmission. Two actions were taken in this regard: a) the Epidemiological Surveillance Emergency Service of Catalonia (SUVEC) recontacted all confirmed cases in Catalonia aged 17–19 years diagnosed after 15 June to identify possible cases not previously identified as part of the outbreak; b) active case-finding, with screening points set up in the urban settings. Local services in Girona, Vallès and Tarragona systematically contacted travel agencies to identify venues and activities where exposure occurred.

**Table 2:** Specific outbreak control measures on investigation of the Menorca trip outbreak of SARS-CoV-2. Catalonia, June, and July of 2021.

Measure	Time	Notes
**Testing and confirmed case isolation**. Performing epidemiological surveys and contact tracing in confirmed cases.	From 15 June to 10 July	Routinely carried out. Cases and their families were informed of how to manage the infection at home.
**Contact tracing**. Identification of close contacts, testing of contacts and establishment of quarantine, if indicated.	From 15 June to 10 July	Cases were asked for their close contacts. These were contacted and informed.
**Surveillance intensification.** Re-contacting all confirmed cases in Catalonia aged 17–19 years diagnosed after 15 June.	24 June	These young people had already been called for contact tracing and the routine survey. The aim was to detect possible cases not previously identified as part of the outbreak.
**Active case-finding**. Screening points set up for testing all identified close contacts and for young people who travelled to Menorca.	From 28 June to 2 July	Screening points were set up in the urban settings: Barcelona, Girona, Figueres and Tarragona. Text messages (SMS) were sent to inform the relevant people of the option of being tested at these screening points.
**Exposures and sources**. Study of transport, accommodation, and activities.	From 23 June to 18 July	On 23 June, the ad-hoc epidemiological survey of Menorca was designed for confirmed cases. This was systematically carried out in confirmed cases from 25 June to 18 July.
**Coordination**. The Barcelona Public Health Agency (ASPB) conducted the outbreak investigation, with the support of the other regional public health services.	From 23 June to 18 July	The different local services identified the cases and carried out the ad-hoc epidemiological survey of Menorca. They reported these cases to the Public Health Agency of Catalonia (ASPCAT) and the ASPB, which analysed the information comprehensively. With the data, a daily update and a census of identified cases and contacts were produced, which were distributed among the local services and to the central services of the ASPCAT.

### Ethical concerns

This outbreak report is exempt from the requirement of approval by an ethics committee because it was an outbreak investigation supported by article 5.b. of Decree 203/2015, on 15 September [15], creating the Epidemiological Surveillance Network and regulating the reporting systems for notifiable diseases and epidemic outbreaks.

## Results

### Description of cases and their close contacts and temporal distribution

We identified 515 primary cases of SARS-CoV-2 with epidemiological links related to the outbreak (Table 3); 296 (57.5%) women and 219 (42.5%) men. We identified 2,280 close contacts: 1,181 (51.8%) women and 1,099 (48.2%) men. The close contacts: primary case ratio was 4:1. Of the close contacts, 245 (10.8%) tested positive for SARS-CoV-2; 131 (53.5%) women and 114 (46.5%) men. The average age was 17.1 years in primary confirmed cases and 18.8 years in secondary cases. By types of exposure of the close contacts, there were 1,466 (64.3%) friends of primary confirmed cases and 699 (30.7%) were household contacts, of whom 211 (14.4%) and 33 (4.7%) tested positive for SARS-CoV-2, respectively. The attack rate was 27.2 per 100 exposed persons, 28.9 in women and 25.3 in men. Regarding clinical course, none of the affected individuals died, required intensive care unit admission, or was hospitalized. Among primary cases, 402 (78.1%) had mild symptoms and 113 (21.9%) were asymptomatic. Among secondary cases, 140 (57.1%) had mild symptoms and 105 (42.9%) were asymptomatic. Whole genome sequencing and analysis was performed in 28 available samples; of these, 18 (64.3%) were identified as B.1.617.2 [16] and 4 (14.3%) as B.1.1.7. In the remaining samples [8], usual deletions in the B.1.1.7 variant were not found.

**Table 3:** Description of confirmed cases and exposure in the Menorca trip outbreak of SARS-CoV-2. Catalonia, June, and July of 2021.

	Women		Men		Total
	N	%	N	%	
**Case criteria **					
Primary case (travelled to Menorca)	296	69.3%	219	65.9%	515
Secondary case (did not travel to Menorca)	131	30.7%	114	34.1%	245
**Age (years) **					
0–12	0	0.0%	0	0.0%	0
13–16	2	0.5%	3	0.9%	5
17–18	413	96.7%	317	95.2%	730
19–25	4	0.9%	9	2.7%	13
26–45	0	0.0%	0	0.0%	0
46–65	8	1.9%	4	1.2%	12
+65	0	0.0%	0	0.0%	0
**Test performed**					
RT-PCR	274	64.2%	235	70.6%	509
RAT	153	35.8%	98	29.4%	251
**Symptoms**					
Symptomatic	316	74.0%	226	67.9%	542
Asymptomatic	111	26.0%	107	32.1%	218
Episode					
First COVID-19	427	100.0%	331	99.4%	758
Reinfection	0	0.0%	2	0.6%	2
**Trip planning** *					
Self-organised	134	45.3%	122	55.7%	256
Travel agency	59	19.9%	37	16.9%	96
No response	103	34.8%	60	27.4%	163
**Accommodation** *					
Tourist apartment rental	138	46.6%	100	45.7%	238
Private houses	26	8.8%	31	14.2%	57
Hotel	16	5.4%	6	2.7%	22
Tourist complex	11	3.7%	9	4.1%	20
Rural house	1	0.3%	2	0.9%	3
No response	104	35.1%	71	32.4%	175
**Attendance at parties** *					
Parties in private apartments	15	5.1%	16	7.3%	31
Parties in Ciutadella’s port	86	29.1%	55	25.1%	141
No response	195	65.9%	148	67.6%	343
**Total **	**427 **	**100.0% **	**333 **	**100.0% **	**760 **

RAT: rapid antigen test; RT-PCR: reverse transcriptase-polymerase chain reaction.

* Data on these variables are only available for the 516 primary cases, who were asked to respond to the Specific Menorca’s Epidemiological survey. On Trip planning 413 (80.0%) young people responded, on Accommodation 412 (79.8%) and on Participation in parties 321 (62.2%). The percentages, in these variables, are calculated on the total number of primary cases.

**Fig 3:**
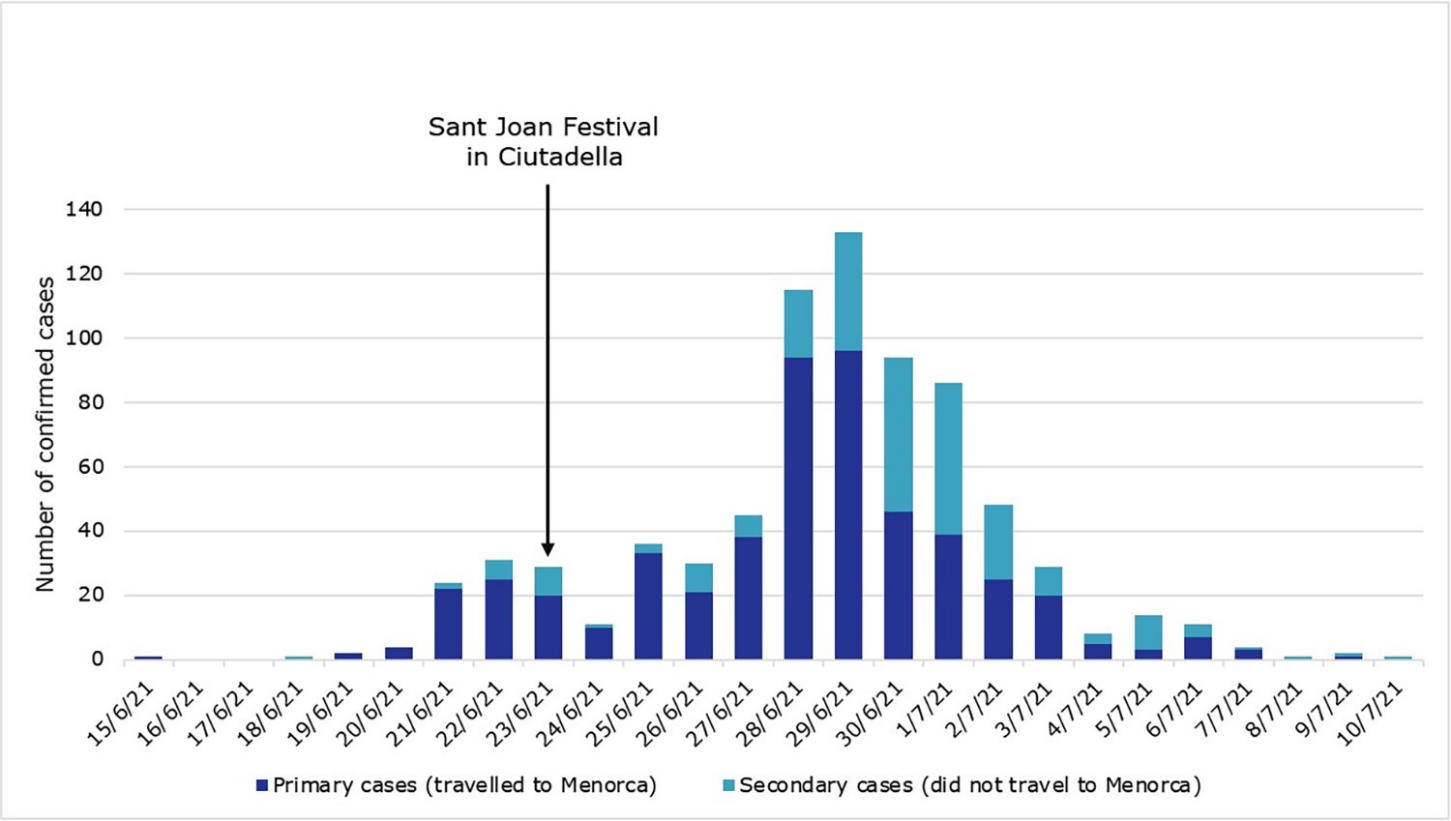
Epidemic curve of the Menorca trip outbreak of SARS-CoV-2, Catalonia, June and July of 2021 (n = 760).

Regarding temporal distribution, the first case was diagnosed on 15 June (Fig 3). Confirmed cases increased until 29 June when the outbreak reached its peak and then gradually declined. The last case was diagnosed on 10 July. The outbreak was over 28 days after the date of diagnosis of the last confirmed case [8]. The episode had a total duration of 45 days. The index case remained unknown due to substantial community transmission.

Among symptomatic cases, the average delay between symptom onset and diagnosis was 1.8 days for primary cases and 1.5 days for secondary cases. Among asymptomatic close contacts, the delay between the last date of contact and diagnosis was 3.0 days.

### Activities possibly encouraging transmission

Trips were principally self-organised by young people (n = 256, 49.7%), but 8 travel agencies arranged a total of 96 (18.6%) trips to Menorca. Most cases rented a tourist apartment (n = 238, 46.2%) or had a private house (n = 57, 11.1%), 141 (27.4) attended parties in Ciutadella’s port and 31 (6.0%) organised or were involved in private parties (Table 3). The parties were described by the attendees during the epidemiological surveys as “crowds of hundreds of people in private villas on different parts of the island, where people did not know each other, and where there was music and alcohol”. These private events were organised by the young people themselves through social networks through “a system of spokespersons and representatives in instant messaging network groups, such as WhatsApp”. The young people reported attending these parties between 12 and 20 June. Young people reported organising themselves through social networks to attend. Youth described the Sant Joan Festival in Ciutadella as a “large binge drinking gathering in the port of the city, followed by parties in the nightlife venues of the area”. Prevention measures for SARS-CoV-2 transmission were reported as not being followed.

Youth reported that they were aware of the various restrictions in Catalonia and Menorca. Interviewed youth stated that they themselves organised the trips through social networks to arrange the travel and accommodation to manage restrictions. One young person stated that “we knew that the maximum number of people who could gather was 10, so we rented adjacent tourist flats to comply with the restriction”.

### Geographical distribution of cases

**Fig 4:**
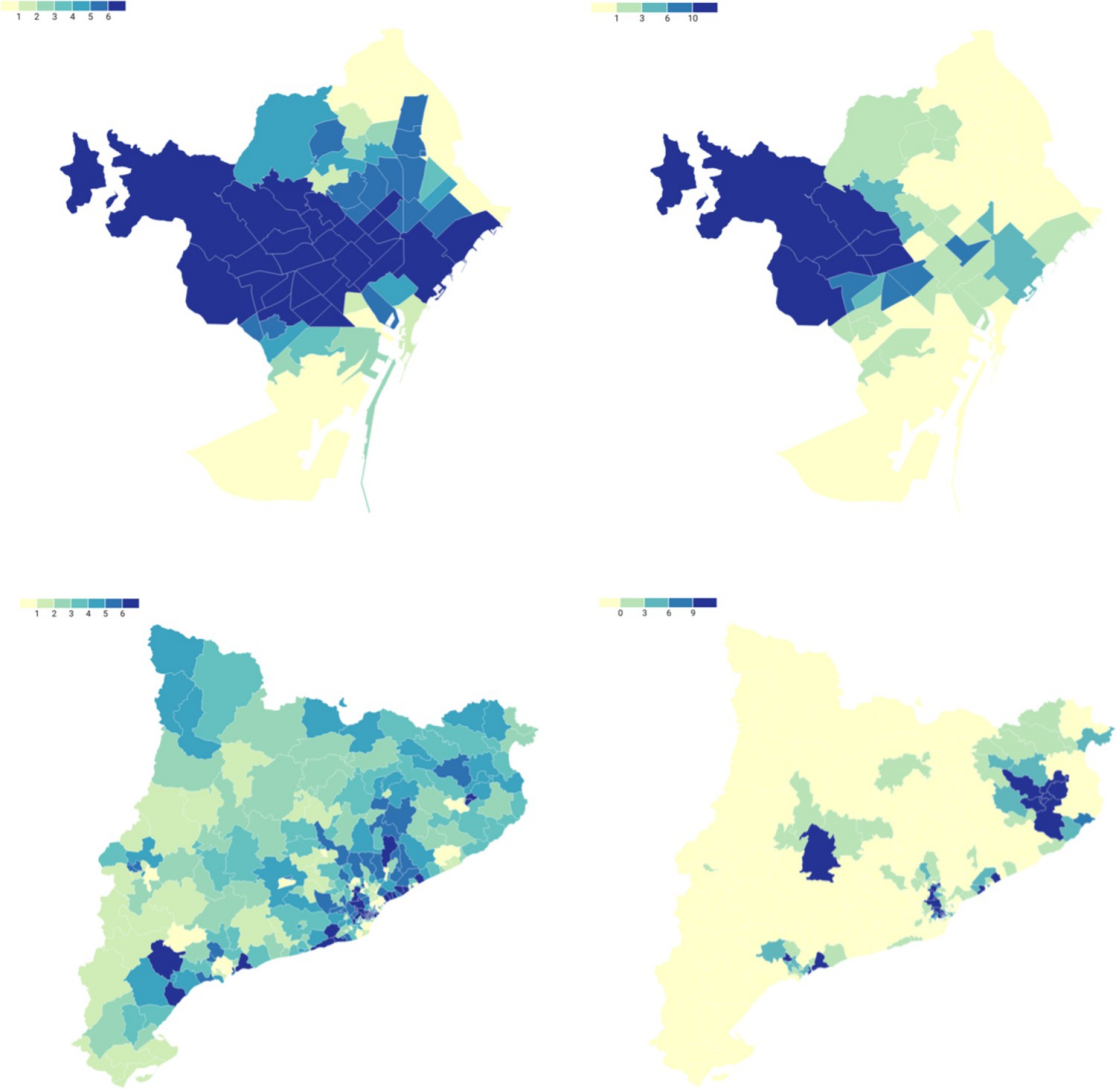
Geographical distribution of the confirmed cases of Menorca trip’s outbreak of SARS-CoV-2 by Basic Health Areas, in Barcelona and Catalonia, June and July of 2021. **a.** Composite socioeconomic index of Basic Health Areas of Barcelona City, in Septiles (1 = most disadvantaged; 7 = most advantaged); **b.** Number of confirmed cases of each Basic Health Area of Barcelona, 15th June– 10th July; **c.** Composite socioeconomic index of Basic Health Areas of Catalonia, in Septiles (1 = most disadvantaged; 7 = most advantaged); **d.** Number of confirmed cases of each Basic Health Area of Catalonia, 15th June– 10th July.

All the confirmed cases were diagnosed after the trip, in Catalonia. Most confirmed cases were concentrated in the city of Barcelona (n = 203, 26.7%). A clear gradient was observed in the city between the CSI of catchment areas and the number of confirmed cases: cases were concentrated in the areas with the highest CSI, particularly the district of Sarrià-Sant Gervasi (Fig 4A and 4B). Likewise, there were fewer cases in the areas with the lowest CSI. Within Catalonia, confirmed cases lived in Girona (n = 95, 12.5%), Sant Cugat del Vallès (n = 64, 8.4%), Sabadell (n = 49, 6.4%) and Vilassar de Mar (n = 32, 4.2%), where the CSI is also high (Fig 4C and 4D). A gradient was also observed between CSI and confirmed cases. Furthermore, an urban-nonurban pattern was found at the local level, as there were few confirmed cases in rural areas of Catalonia, except for the village of Guissona, where there was a large number of cases (n = 15, 2.0%). There was a significative positive spatial autocorrelation between CSI and confirmed cases (bivariate Moran’s I: 0.171; p value <0.005).

**Fig 5:**
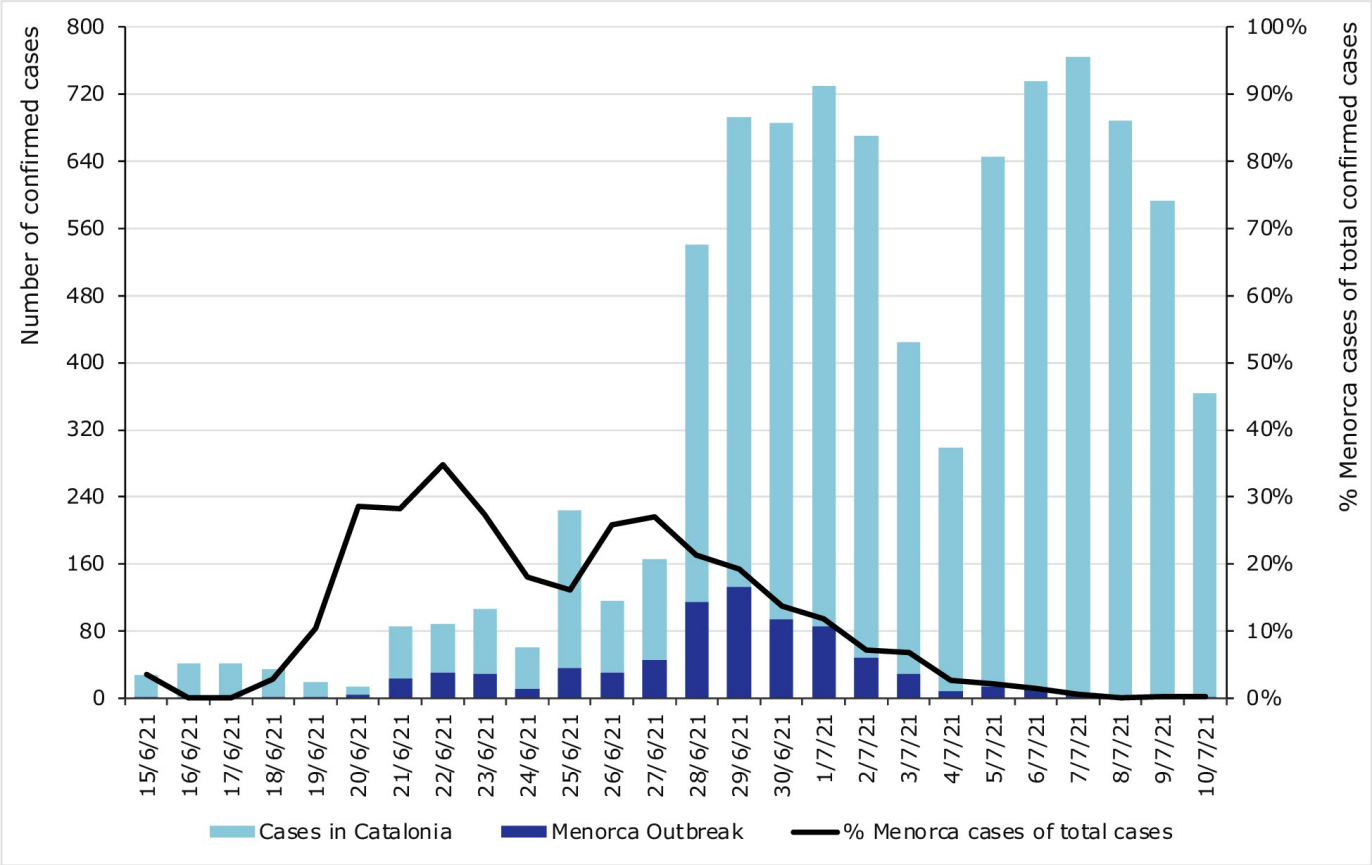
Number of confirmed cases of Covid-19 aged 17–19 in Catalonia during Menorca outbreak and percentage of confirmed cases related with Menorca’s outbreak, Catalonia, June and July of 2021.

Cases related to this outbreak made up 9% of the confirmed cases aged 17–19 years in Catalonia between 15 June and 10 July. In the days surrounding the Sant Joan festival, the percentage of confirmed cases linked to the Menorca outbreak remains between 25–30% of the total number of confirmed cases in this age group (Fig 5). In some clusters of Basic Health Areas this percentage exceeds 50% of the total confirmed cases aged 17–19 years. This is particularly relevant in Sant Cugat del Vallès, Sarrià-Sant Gervasi and Les Corts, areas with high SCI.

## Discussion

This study describes an outbreak related to trips to Menorca by secondary school students in June, 2021. We identified 760 confirmed cases of SARS-CoV-2. A large COVID-19 outbreak with a high attack rate is a public health challenge. Based on the characteristics of the delta variant [17] and its high COVID-19 incidence and dominance in the area, social activities probably played an important role in the transmission. We would like to highlight some elements:

### 1. Prevention and management of COVID-19 outbreaks in advantaged socioeconomic contexts

In Barcelona there are social inequalities in the cumulative incidence of COVID-19 [18]. Disadvantaged neighbourhoods have significantly higher incidences from the first to the fourth waves [19, 20]. This outbreak did not follow the socioeconomic pattern previously identified in the city, as it affected mainly young people from advantaged socioeconomic backgrounds. New outbreaks could appear in economically advantaged settings. The lessons learned from this outbreak will be key to managing them: knowledge of age and social class are crucial to establish an approach strategy.

### 2. Risk perception

As the investigation of this outbreak noted, young people were not aware of COVID-19, and preventive measures were relaxed. In Europe, it has been observed that age also influences risk perception [21] and contribute to the relaxation of preventive measures [22]. The Balearic Islands is one of the main tourist destinations in Europe [23] where people from different countries enjoy their holidays. However, the motive for some of these trips is to drink alcohol and attend parties. Therefore, it is not easy to introduce regulations or limitations to avoid SARS-CoV-2 transmission in these places. In addition, some confirmed cases flew to their usual residence with infective capacity [24], and SARS-CoV-2 outbreaks have previously been reported in aeroplanes [25]. This outbreak raises the need to better understand COVID-19 risk perception.

### 3. The challenge of outbreaks arising from super dissemination events

Participation in mass events has previously been associated with an increased risk of COVID-19 infection: a relative risk of 2.5 was reported in Catalonia for attending mass music events in the same time period as this outbreak [26], and a prior study reported an odds ratio of 2.4–3.9 for going to bars and restaurants [27]. However, transmission could have been considerably reduced by correct use of masks, even in a pre-vaccination scenario such as this one [28]. The informal organisation of travel and parties and the lack attendance lists increased the difficulty of tracing contacts and identifying sources of infection, requiring extensive testing measures. Anticipating these outbreaks in mass events and preparing systems is one of the major lessons from this outbreak. We know that in summer there will be trips and festivals, and in winter there will be ski breaks.

### 4. This outbreak highlights the need to establish a clear line of leadership and rapid and effective coordination measures between institutions, authorities and stakeholders from different regions and services

We would like to highlight the role of interdisciplinary teams and networking allowed for a rapid control of the outbreak. This specific team processed and updated the outbreak data daily, provided indicators and drew up a daily update for public communication. Furthermore, sharing information and discuss, where the different exposures detected and the activities that favoured transmission were analysed to allow the implementation of rapid and effective measures. Explosive outbreaks related to tourism may occur, and a coordinated response is required among all the stakeholders involved where the outbreak occurs and the place of residence of the cases. Early detection of these outbreaks enables action to be taken in transmission settings. To our knowledge, there are few prior publications on strong coordination in response to a COVID-19 outbreak.

### 5. Impact of this outbreak

With the information we currently have, it would be extremely bold to claim the specific impact of this outbreak on the transmission dynamics of Covid-19 in Catalonia in the summer of 2021. In the first place, although this is possibly the largest outbreak of Covid-19 documented in Catalonia, it is not the only big outbreak in the summer of 2021. Other outbreaks that were reported then are now being analysed. Secondly, while many chains of transmission were successfully interrupted, it is likely that many others were not. Therefore, taking into account the subsequent evolution of Covid-19, data would suggest that this outbreak contributed to the explosiveness of the fifth wave of Covid-19 in Catalonia (between 13 June and 1 October).

### Limitations

A limitation of the study was selection bias. We could not obtain a record of all the young people who had participated in the trip. Individuals were identified from epidemiological surveys and primary care. Another limitation is the number of young people who refused to provide data to the public health services about their close contacts and the activities undertaken during their trip. This could be related to the socioeconomic status of the related cases.

From our experience, we strongly recommend identifying, through stakeholder mapping [29], the stakeholders who will be able to contribute to the management of large outbreaks. Strengthening relationships could facilitate a quicker and more effective response in these situations. We recommend addressing preventive measures for youth to modify their risk perception, through specific communication campaigns [30].

## Conclusion

This study is one of the first to address the problem of massive SARS-CoV-2 outbreaks in European tourist destinations. A total of 760 people were affected, aged 17–19 years, who had taken a trip to Menorca. Transmission may have been encouraged by the relaxation of preventive measures in the festive environment and during holidays. Rapid identification of cases and close contact tracing allowed interruption of transmission chains and rapid control of the outbreak. The establishment of a clear line of leadership and a strong coordination structure allowed continuous communication between institutions. Interdisciplinary teams facilitated a comprehensive approach to the outbreak and its investigation. Stakeholder relationships are key to managing complex COVID-19 outbreaks.

## Supporting information

S1 Dataset Confirmed cases of Menorca’s outbreak. (CSV)

S2 Dataset Close contacts of confirmed cases of Menorca’s outbreak. (CSV)

S1 Appendix Epidemiological survey. Covid 19 case notification survey.
(PDF)

S2 Appendix Ad-hoc epidemiological survey of Menorca’s outbreak. (PDF)

S1 Table Additional data from confirmed cases. (XLSX)

S2 Table Additional data from close contacts. (XLSX)
