# Supplementary material for: A large outbreak of COVID-19 linked to an end of term trip to Menorca (Spain) by secondary school students in summer 2021
Source: PLoS One. 2023 Feb 3;18(2):e0280614. doi: 10.1371/journal.pone.0280614 (PMC9897546; doi:10.1371/journal.pone.0280614)
Supplement: S1 Appendix — Covid 19 case notification survey. (PDF) [file pone.0280614.s003.pdf]

Epidemiological Survey
Covid 19 case notification survey

1. Identifying information:

Name and surname: ____

Sex: Male ☐ Female ☐ NS / NC ☐

Date of birth: __ __ / __ __ / __ __ __ __

Age (in years): ____ (if no date of birth is available)

Age in months (if less than 2 years old) ____

CIP _________ ID / NIE / PASSPORT: _________

Address: _________ Telephone: _________

E-mail address: ____________________

Place of residence

Postcode: ______ Municipality: _______

2. Type of residence:

Single ☐ Residential centre ☐ Foster flat ☐ Holiday home ☐

With how many persons do you share the household? ____

Lives alone: Yes ☐ No

Number of cohabitants excluding the respondent: ____

Support. Do you know someone who can help you or give you the support you need during isolation? Yes ☐ No ☐

Do you have at-risk household members (grandparents, immunocompromised, etc.) Yes ☐ No ☐

Do you have a minor, elderly or dependent person in your care? Yes ☐ No ☐

Are there are any problems of cohabitation or other difficulties at home? Yes ☐ No ☐

Do you have guaranteed access to shopping, food, medicines, social support? Yes ☐ No ☐

3. Language / cultural difficulties:

Language barrier: Yes ☐ No ☐

Cultural barrier: Yes ☐ No

Ability to understand isolation measures Yes ☐ No ☐

4. Clinical data

Date of consultation: (day / month / year) _ _ / _ _ / _ _ _ _

Symptoms YES ☐ NO ☐

If yes, give date of onset of symptoms (day / month / year) _ _ / _ _ / _ _ _ _ _

☐ Cough (dry)

☐ Fever or recent history of fever

☐ Shortness of breath (dyspnoea)

☐ General malaise

☐ Vomiting and/or diarrhoea ☐ Vomiting and / or diarrhoea

☐ Loss of smell and / or taste

☐ Sore throat (onidophagia)

☐ Headache

☐ Loss of appetite (anorexia)

☐ Myalgia and / or arthralgia

☐ Other

5. Reinfection

Date of onset of new symptoms (day / month / year): _ _ / _ _ / _ _ _ _

First confirmed infection ☐

Suspected ☐ Probable ☐ Confirmed ☐

6. Health personnel working in: (single answer)

Health centre ☐

Social-health centre ☐

Other establishments ☐

Specify the type of facility: ____________________

No health worker ☐

Last day of work: _ _ / _ _ / _ _ _ _

Name of the work centre: _____________

Address ____________

Task to be performed__________________

7. Vaccination:

Vaccinated Yes ☐ No ☐

First dose: Pfizer / BioNTech ☐ Moderna ☐ Oxford / AstraZeneca ☐

First dose date: _ _ / _ _ / _ _ _ _

Second dose: Pfizer / BioNTech ☐ Moderna ☐ Oxford / AstraZeneca ☐

Date of second dose: _ _ / _ _ / _ _ _ _

8. Non-health worker working in: (single answer)

Work setting other than health care but with many contacts (many people at the same time or interacting with many people individually) Yes ☐ No ☐

Last day of work: _ _ / _ _ / _ _ _ _

Name of workplace: _____________

Address ____________

9. Scope of possible exposure in the previous 14 days

(Single answer) Health ☐ School ☐ Social ☐ Social-health care ☐ Leisure ☐ Home ☐ Occupational ☐ Other ☐ Unknown ☐

Possible date of exposure: _ _ / _ _ / _ _ _ _

Name of possible place of exposure: _____________

Address ____________

10. Case imported from another country

Yes ☐ No ☐

Specify country: ___________________

Date of arrival in Catalonia: _ _ / _ _ / _ _ _ _

11. Contact with a known confirmed case in the last 14 days.

Yes ☐ No ☐

Setting in which, in the opinion of the person assessing the case, transmission of the infection has occurred: _________

Contact details of the confirmed case:

Names and surnames: ____________________

IPC (not survey): __________________________

Telephone: ____________________________

Date of last contact: _ _ / _ _ / _ _ _ _

Time: ___: ___

Place (name and specific physical address) or grouping (flight, coach): ___________

Context / Setting Health ☐ School ☐ Social ☐ Social-health care ☐ Leisure ☐ Home ☐ Occupational ☐ Other ☐ Unknown ☐

12. Date of diagnosis

(Day / month / year) _ _ / _ _ / _ _ _ _

13- Positive diagnostic tests

RT-PCR / Molecular Techniques ☐

ELISA / CLIA / ECLIENT ☐

Rapid antibody test (RAT) ☐

Antigen detection ☐

14. Sequencing

Random Yes ☐ No ☐

Suspicion of reinfection ☐

Epidemiological link with high-prevalence sites ☐

Infection in properly vaccinated person ☐

Increased incidence ☐

Break in amplification of S gene ☐

Outbreak in animals with human infection ☐

15. Is there isolation?

Can work from home: Yes ☐ No ☐

Cannot work from home and cannot take sick leave: Yes ☐ No ☐

You have to take sick leave: Yes ☐ No ☐

16. Identification of contacts

For each close contact, the following should be collected:

Names and surnames: ___________________________________

Telephone number: _______________________

Date of last contact: _ _ / _ _ / _ _ _ _

Time of contact: __: __

Place (name and specific physical address) or grouping (flight, coach): ____________

Context / Setting Health ☐ School ☐ Social ☐ Social-health care ☐ Leisure ☐ Home ☐ Occupational ☐ Other ☐ Unknown ☐

17. Plotting

Record as many times as there were sites / groupers in the 14 days prior to the onset of symptoms (person tracing):

Date: _ _ / _ _ / _ _ _ _

Time: __: __

Place (exact address) or grouping (e.g. flight, train, coach ...). Enter data in the tool: ____

Context / Setting Health ☐ School ☐ Social ☐ Social-health care ☐ Leisure ☐ Home ☐ Occupational ☐ Other ☐ Unknown ☐

18. Are you affiliated in a transmission chain?

Yes ☐ No ☐

19. Clinical course

Hospital admission Yes ☐ No ☐

Date of hospital admission _ _ / _ _ / _ _ _ _

Date of discharge _ _ / _ _ / _ _ _ _

ICU admission Yes ☐ No ☐

Date of admission to ICU _ _ / _ _ / _ _ _ _

Date of discharge from ICU _ _ / _ _ / _ _ _ _
