# Supplementary material for: A large outbreak of COVID-19 linked to an end of term trip to Menorca (Spain) by secondary school students in summer 2021
Source: PLoS One. 2023 Feb 3;18(2):e0280614. doi: 10.1371/journal.pone.0280614 (PMC9897546; doi:10.1371/journal.pone.0280614)
Supplement: S2 Appendix — (PDF) [file pone.0280614.s004.pdf]

Ad-hoc epidemiological survey of Menorca
Appendix to Covid 19 case notification survey

1. Personal Identification Code: ______________________________

2. Name and surname: ______________________________

3. Municipality of residence: ______________________________

4. Local Epidemiological Service according to municipality of residence:

City of Barcelona ☐ Barcelonès Nord – Maresme ☐ Barcelona Sud ☐ Vallès ☐ Catalunya Central ☐ Girona ☐ Lleida ☐ Tarragona ☐ Ebre ☐

5. Date of the test:

_ _ / _ _ / _ _ _ _

6. Type of test:

RT-PCR ☐ RAT ☐

7. Secondary School: ______________________________

8. Have you travelled to Menorca after 11 June?

Yes ☐ No ☐

9. Have you travelled to Mallorca after 11th June?

Yes ☐ No ☐

10. Number of close contacts in the trip:

_ _

11. Copy and attach here the list of close contacts on the trip from the epidemiological survey:

12. Did you go to a travel agency?

Yes ☐ No ☐

Name of place: ____

Address: ____

Telephonic contact: ____

13. Accommodation in which you have stayed, as accurate as possible

Tourist appartment rental ☐ Private house ☐ Hotel ☐ Turist complex ☐ Rural house ☐ Don't Know/Don't Answer ☐

Name of place: ____

Address: ____

Telephonic contact: ____

14. In which activities did you participate in Menorca?

Organised activities for tourists ☐ Parties in private homes ☐ Sant Joan festivities in Ciutadella ☐ Pubs and restaurants ☐ Others ☐

Name of place: ____

Address: ____

Telephone contact: ____

15. Observations
